# Supplementary material for: Oral Immunization with rVSV Bivalent Vaccine Elicits Protective Immune Responses, Including ADCC, against Both SARS-CoV-2 and Influenza A Viruses
Source: Vaccines (Basel). 2023 Aug 23;11(9):1404. doi: 10.3390/vaccines11091404 (PMC10534613; doi:10.3390/vaccines11091404)
Supplement: Supplementary file 1 [file vaccines-11-01404-s001.zip › vaccines-2551358-supplementary.pdf]

Supplemental Figure S1. The diagram of the vaccine candidate rVSV-EM2-SP_{Delta} structure (derived from Ao et al. J. Viol. 2022)

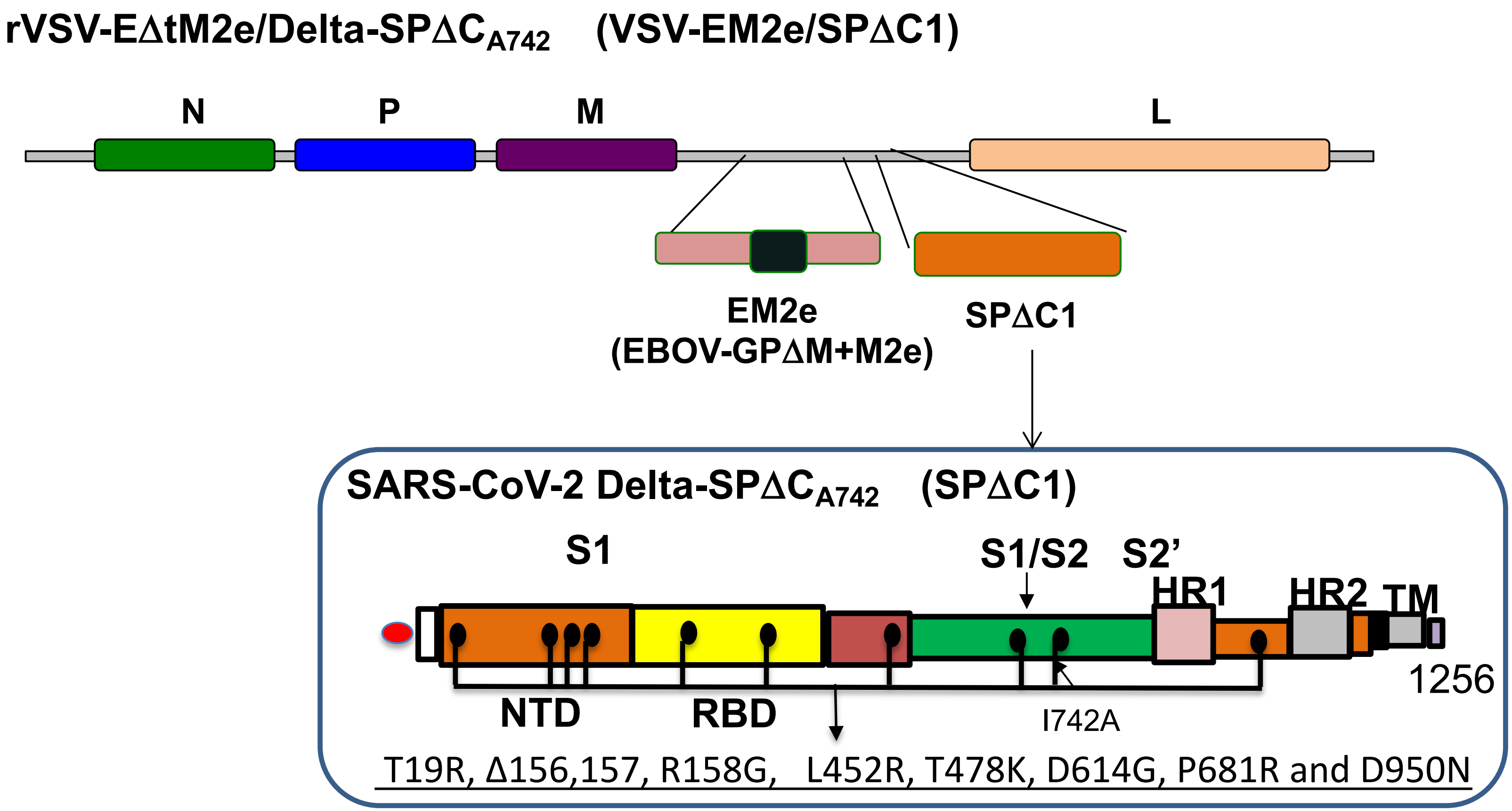

Supplemental Figure S2. Expression of SPΔCWT, SPΔCDelta, and Hu-IAV-M2 in 293T cells.

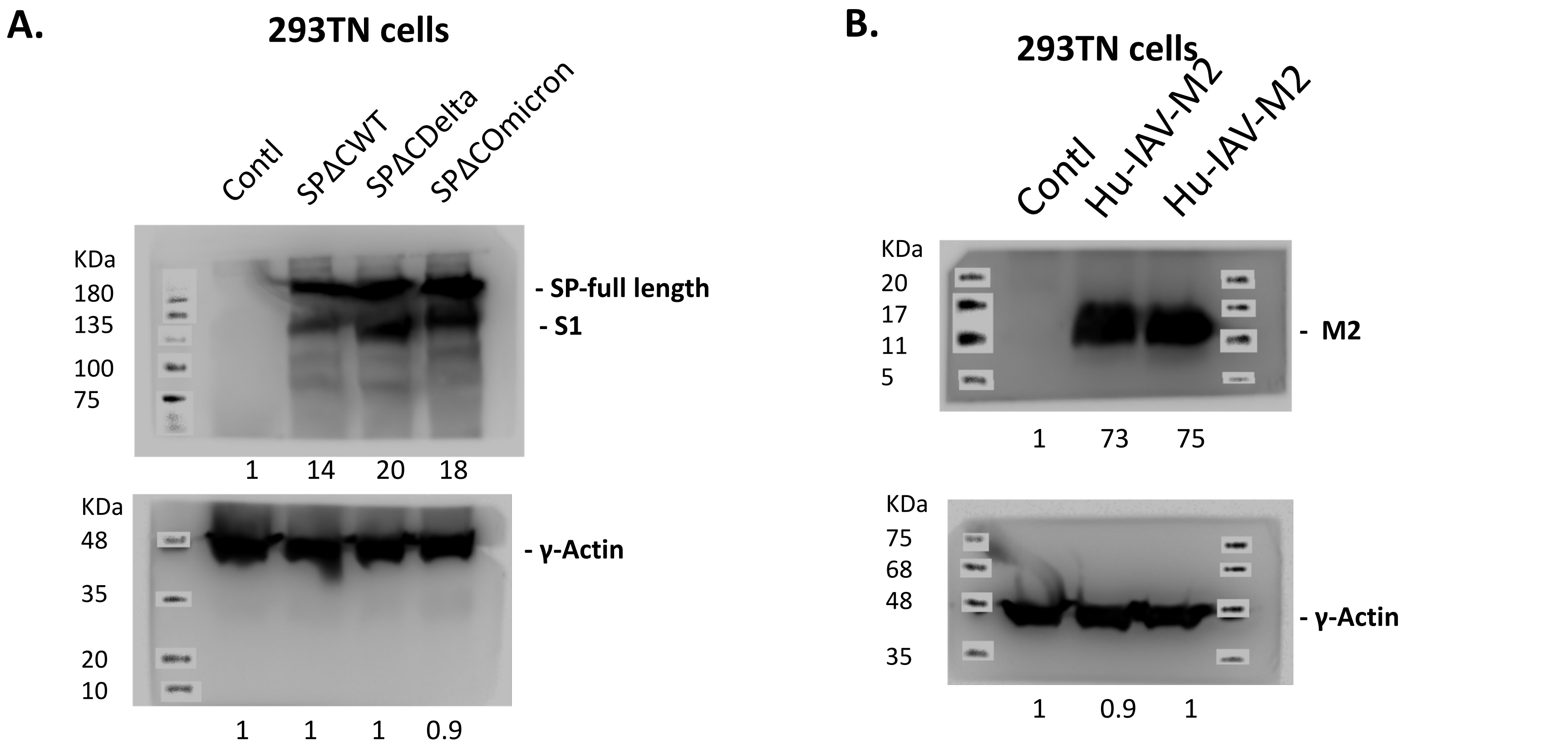

Suppl Figure S2: The 293TN cells were transfected with plasmids pCAGGS-SARS-CoV-2 SPΔC (deleted C-terminal 17 aa) from the original strain Wu-Han-1 (SPΔC_{WT}), SPΔC_{Delta}, or SP$\Delta C_{Omicron}$ (A), or plasmids pCAGGS-Hu-IAV-M2 (B). After 48 hrs of transfection, the transfected 293T cells were lysed in RIPA buffer, and directly loaded into the 12 % SDS–PAGE gel and the presence of each of viral proteins was detected by WB with various corresponding antibodies and the detected proteins were indicated in the right side of the each gel.
